# Supplementary material for: A study paradigm integrating prospective epidemiologic cohorts and electronic health records to identify disease biomarkers
Source: Nat Commun. 2018 Aug 30;9:3522. doi: 10.1038/s41467-018-05624-4 (PMC6117367; doi:10.1038/s41467-018-05624-4)
Supplement: Supplementary file 1 — Supplementary Information [file 41467_2018_5624_MOESM1_ESM.pdf]

Description of Additional Supplementary Files

File Name: Supplementary Data 1
Description: List of all pheWAS associations with Bonferroni $p<0.05$.

File Name: Supplementary Data 2
Description: List of all pheWAS associations with FDR $q<0.1$.

File Name: Supplementary Data 3
Description: Skewness values for the PheWAS association statistics for each ARIC biomarker phenotype.

File Name: Supplementary Data 4
Description: ARIC biomarker phenotypes used in the analyses.
