# Supplementary material for: A study paradigm integrating prospective epidemiologic cohorts and electronic health records to identify disease biomarkers
Source: Nat Commun. 2018 Aug 30;9:3522. doi: 10.1038/s41467-018-05624-4 (PMC6117367; doi:10.1038/s41467-018-05624-4)
Supplement: Supplementary file 3 — Description of Additional Supplementary Files [file 41467_2018_5624_MOESM3_ESM.pdf]

Supplementary Information

A study paradigm integrating prospective epidemiologic cohorts and electronic health records to identify disease biomarkers

Mosley et al.

Supplementary Figure 1

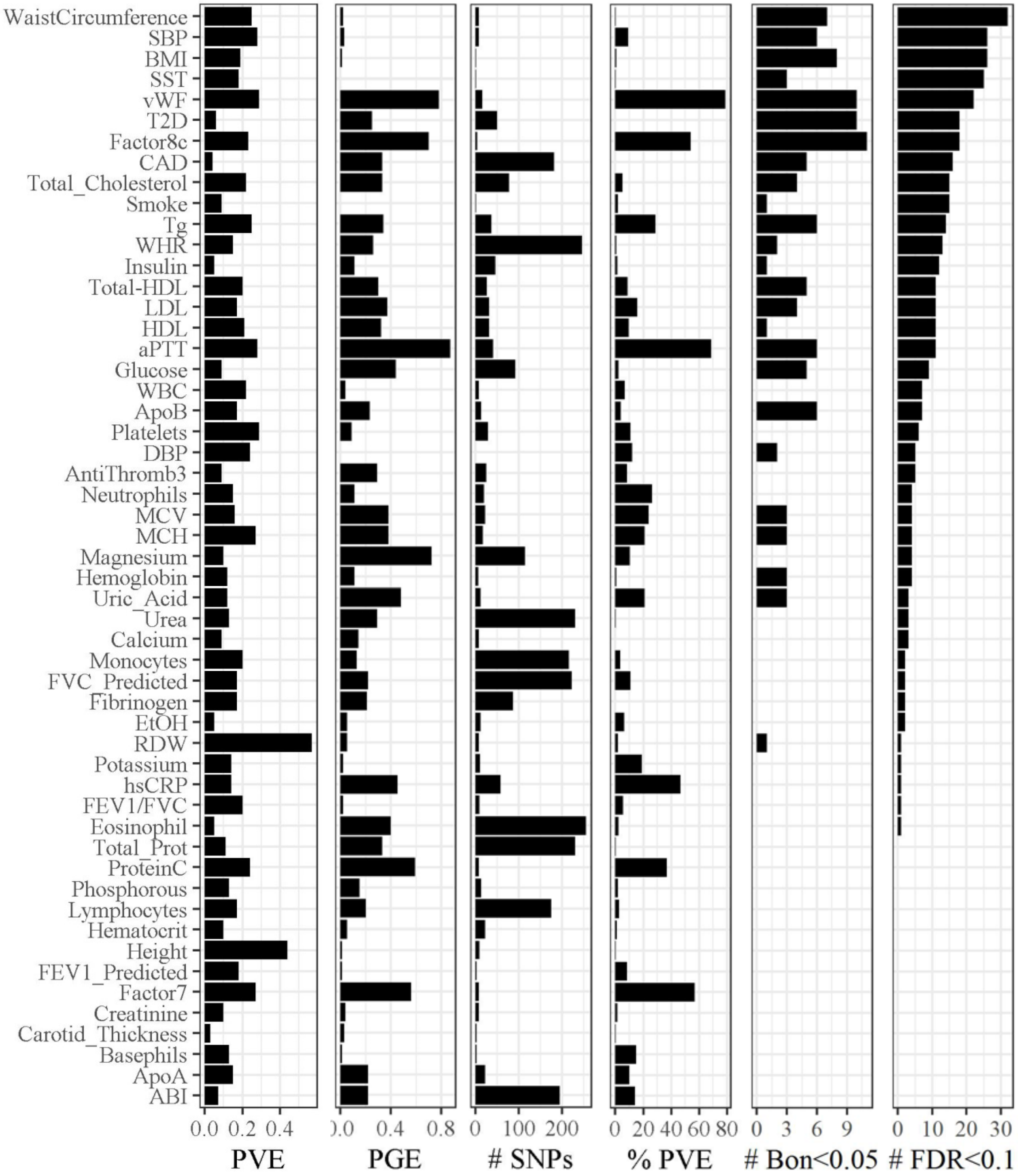

Supplementary Figure 1: Summary of BSLMM analyses on the 53 ARIC biomarkers. For each biomarker, the histograms show the estimate of the proportion of phenotypic variance explained (PVE), the proportion of genetic variance explained by SNPs with large effects (PGE), the estimated number of large-effect SNPs modulating the biomarker (# SNPs), the proportion of the PVE explained by the genetic predictor of the biomarker level (% PVE), the number of PheWAS assocation with Bonferroni p<0.05 and the number of associations with FDR q<0.1. An asterisk (*) indicates the %PVE was not determined (for binary phenotypes). Biomarkers are sorted by the number of associations with FDR q<0.1 with the PheWAS phenotypes (shown in the last column).

Supplementary Figure 2 (part 1)

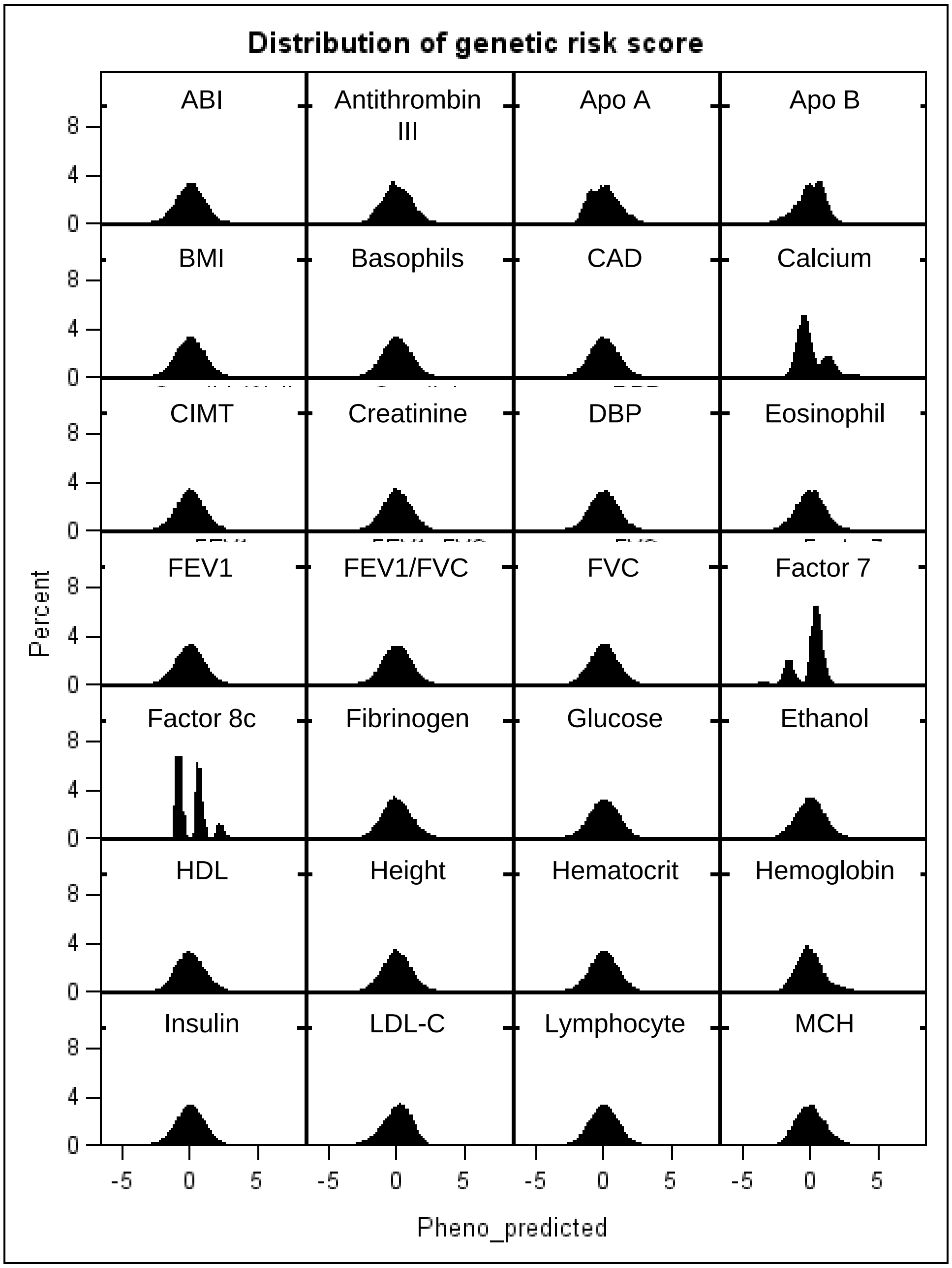

Figure and legend continued on next page.

Supplementary Figure 2 (part 2)

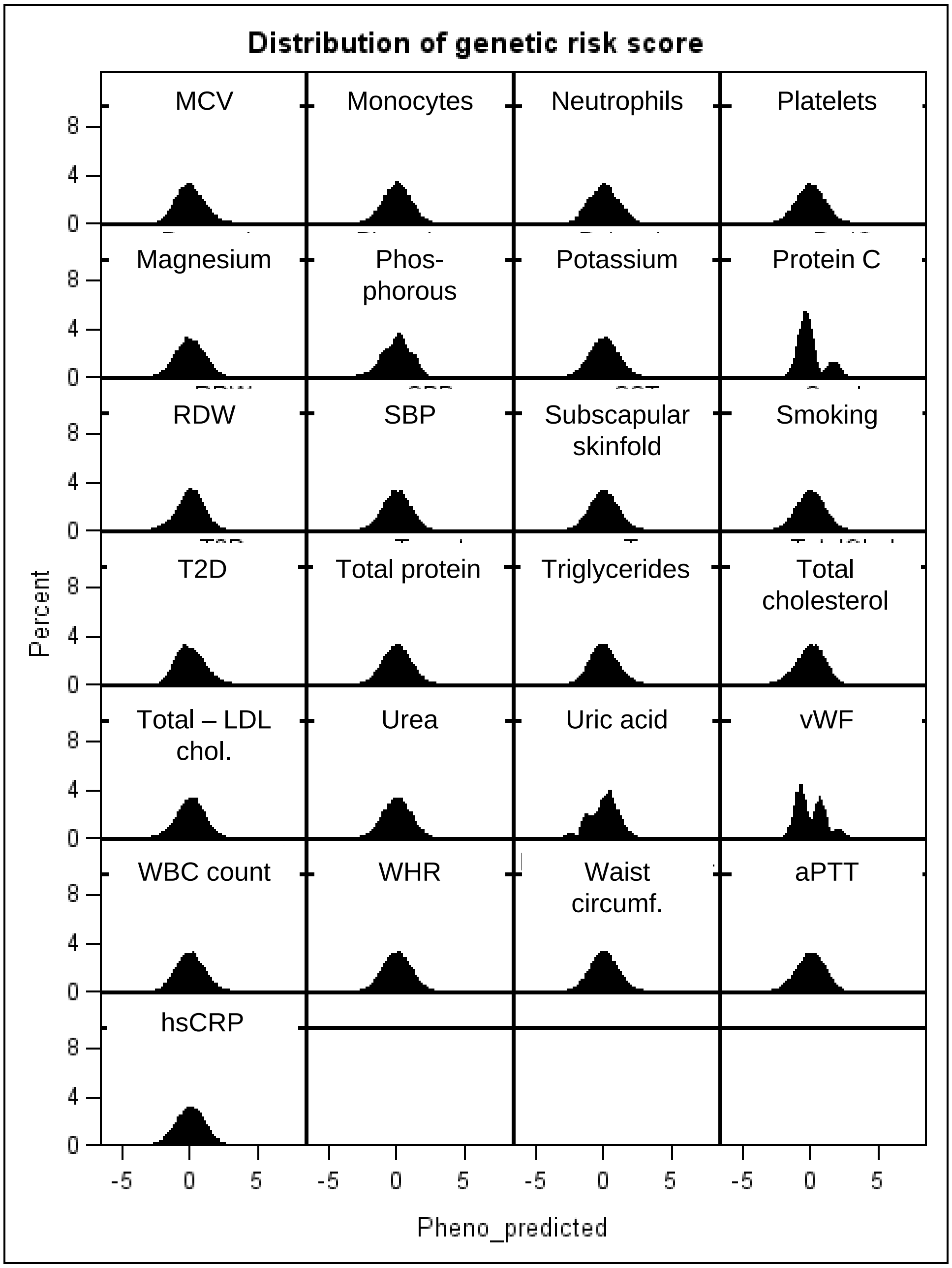

Supplementary Figure 2: Frequency distributions for each of the 53 genetically predicted ARIC biomarkers in the EHR population. Each panel represents the distribution for a genetically predicted biomarker within the EHR population. Genetically predicted biomarkers were computed using SNP weights estimated from BSLMM analyses of each ARIC biomarker. The x-axis units are standard deviations from the mean.

Supplementary Figure 3

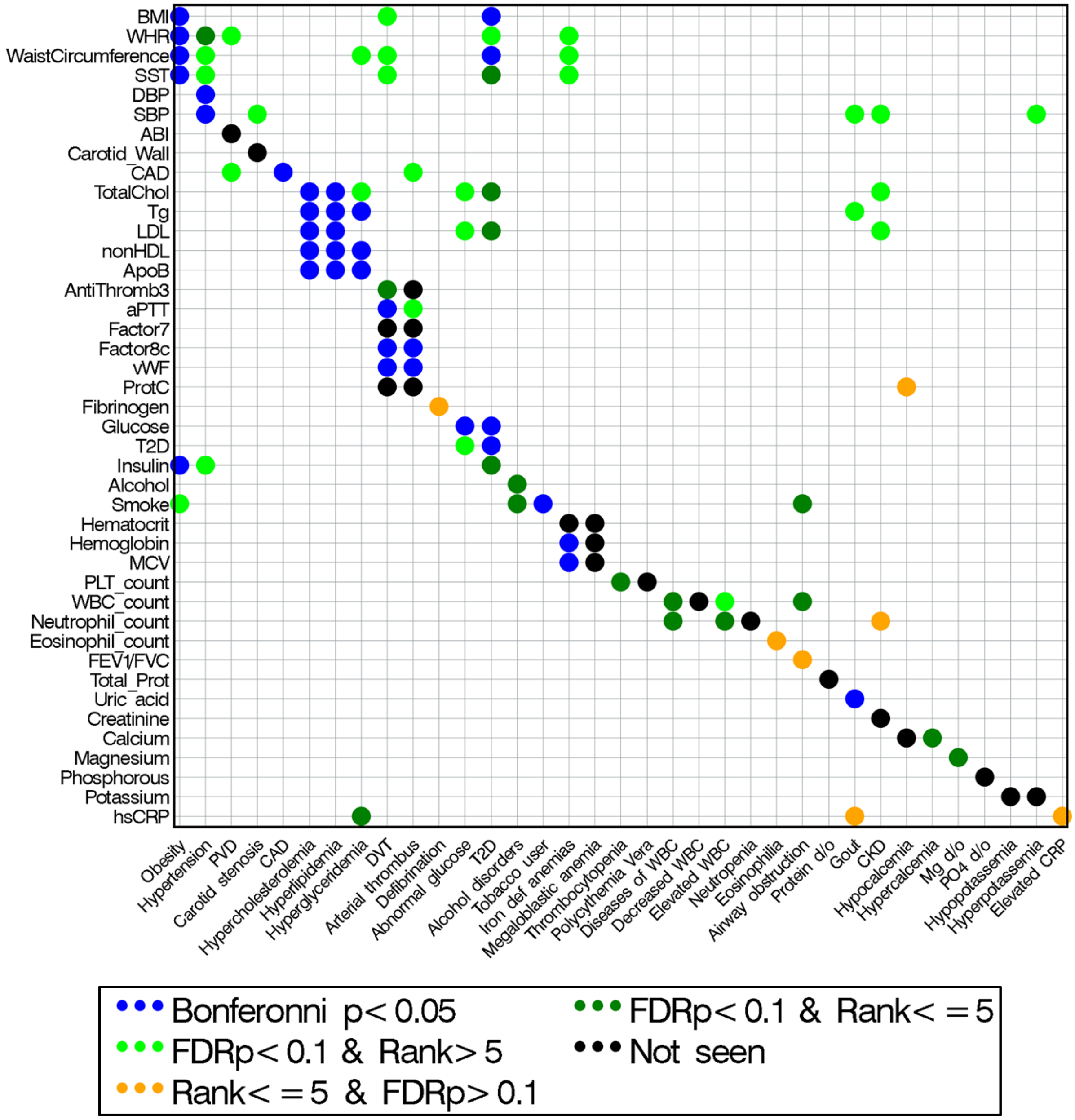

Supplementary Figure 3 Associations with positive controls. Positive control phenotypes were identified *a priori* for 42 ARIC biomarkers. The scatter plot shows associations between the positive control phenotypes (x-axis) and the 42 genetically predicted biomarkers (y-axis). Colors denote whether the significance of the association meets Bonferroni p-value, FDR p-value threshold and rank order criteria. A black dot denotes positive control pairs where an expected association met none of the association criterion.

Supplementary Figure 4

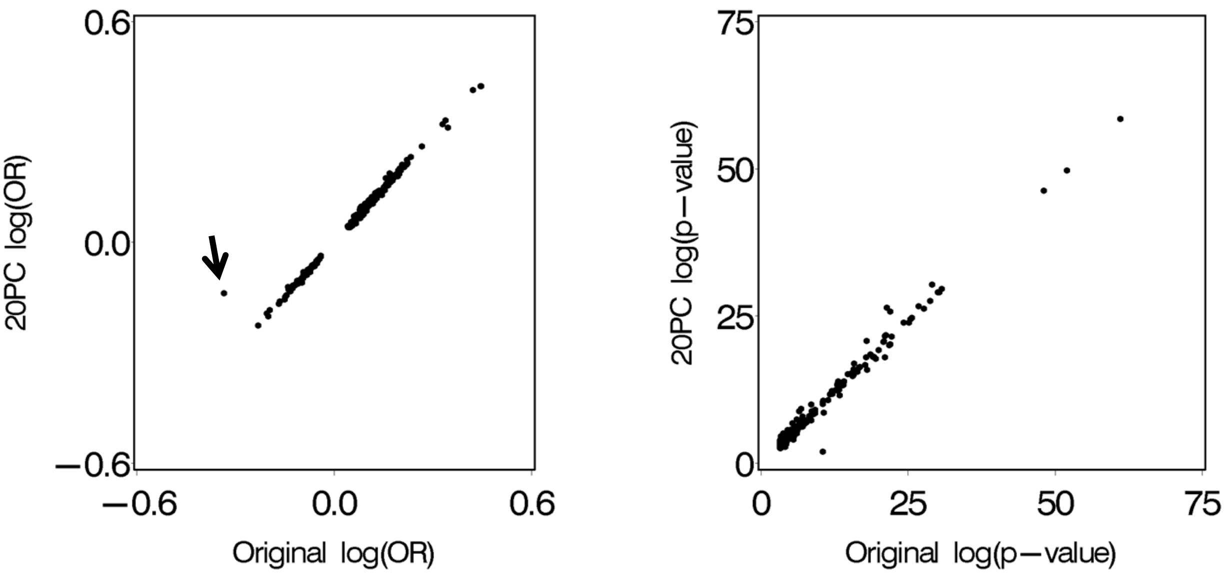

Supplementary Figure 4: Comparison with adjusting for 20 PCs. The scatter plots compare log(odd-ratio) [left panel] and log(p-value) [right panel] for associations with FDR q<0.1 in the primary results to results obtained when adjusting both the BSLMM and logistic regression models for 20 PCs. The arrow points to an outlying association between the “red blood cell distribution width (RDW)” biomarker and the phenotype “Disorders of iron metabolism”.

Supplementary Figure 5

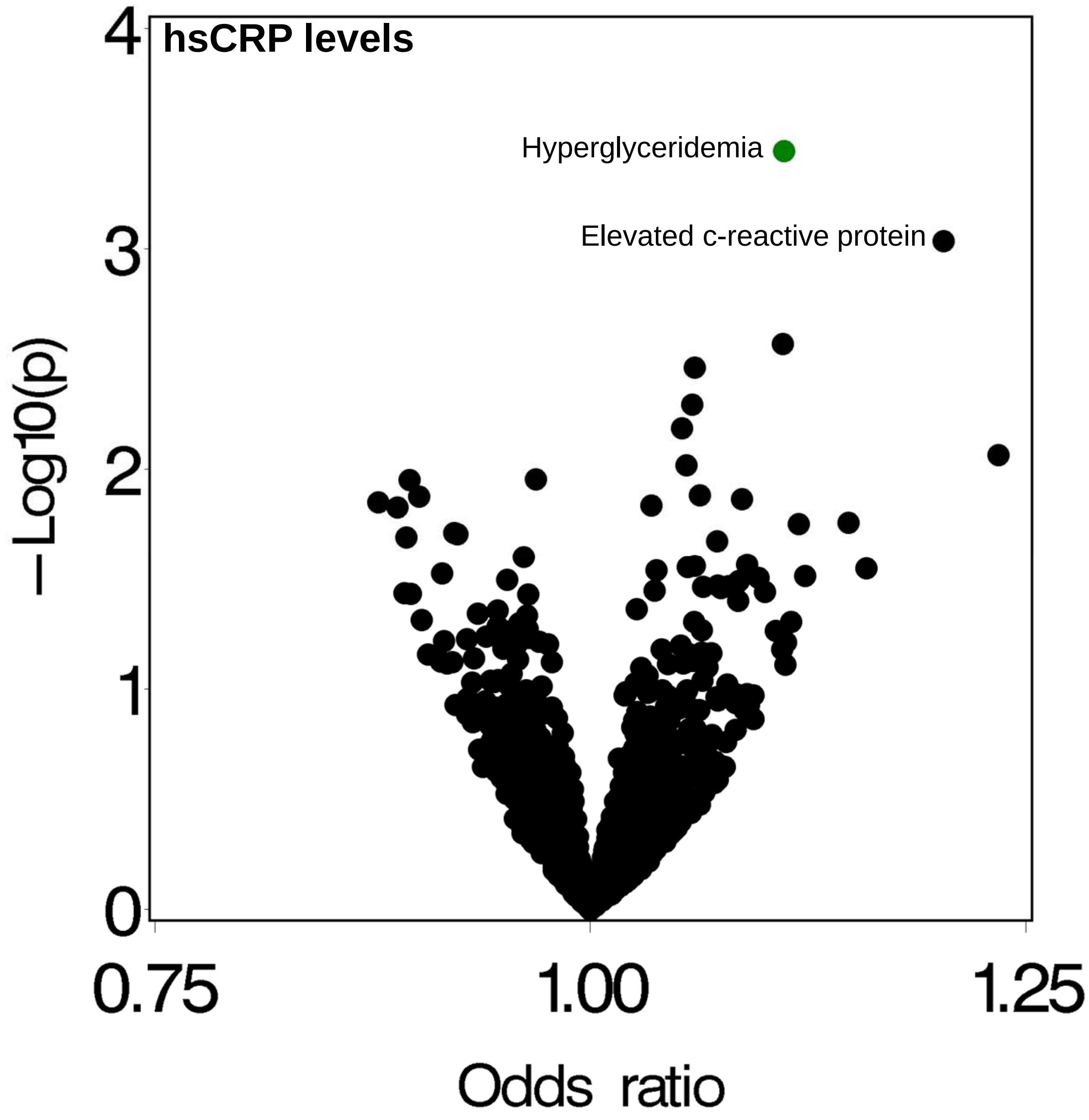

Supplementary Figure 5: Associations with hsCRP. The scatter plot summarizes pheWAS analyses for a genetic predictor of high sensitivity C-reactive protein (hsCRP). Odds ratios are from logistic regression analyses, adjusting for birth decade, gender and 3 principal components, and represent the risk associated with a 1 standard deviation change in the value of the genetic predictor. Points highlighted in green are significant at FDR q<0.1.

Supplementary Figure 6

ARIC Biomarkers

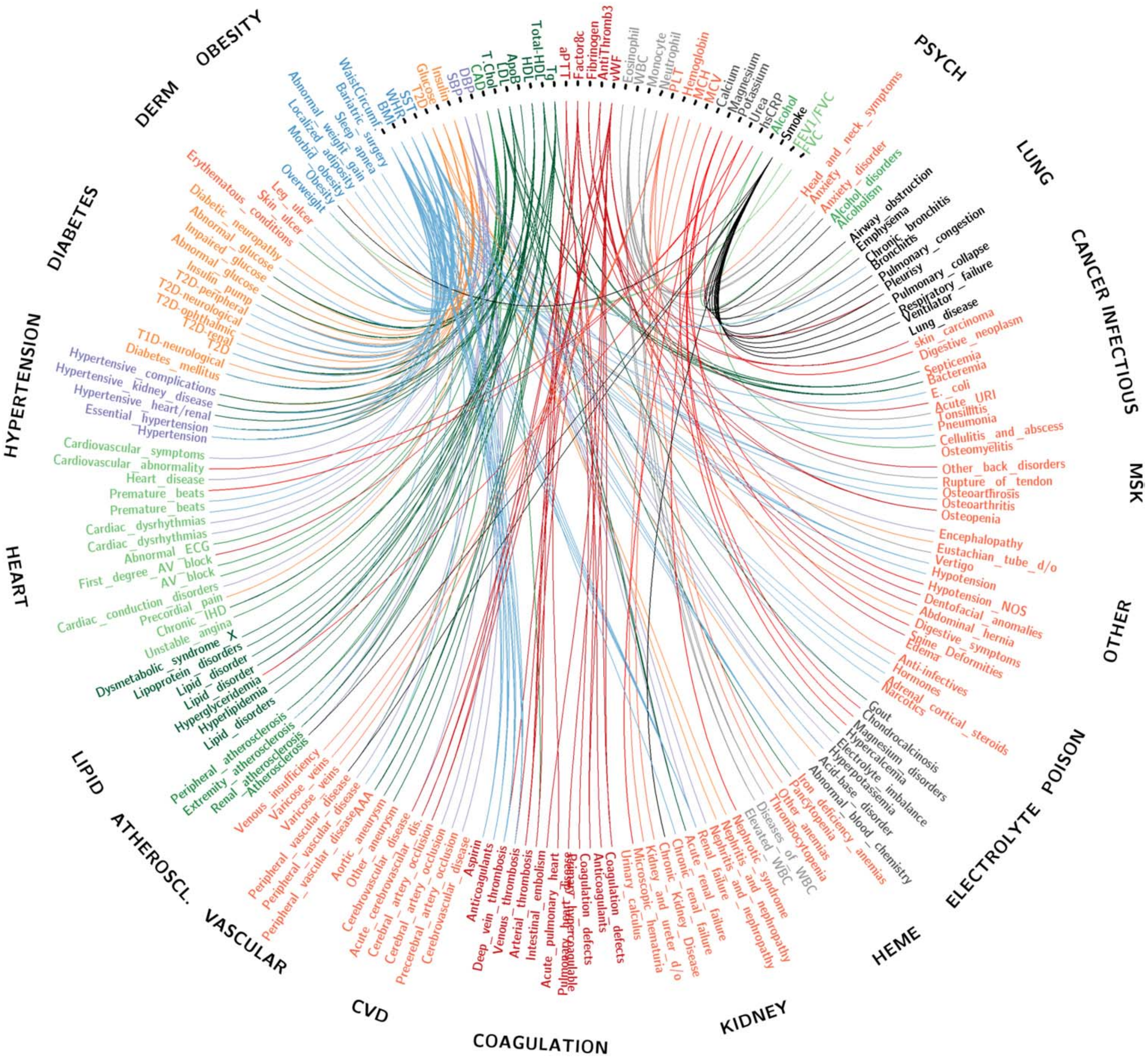

Supplementary Figure 6: Associations with FDR p<0.1 and Bonferroni p>=0.05. Circos plot showing 261 associations between the genetic predictors of the ARIC biomarkers and pheWAS phenotypes. Associations are denoted by lines. Coloring is used to highlight similar groups of biomarkers and pheWAS phenotypes.

Supplementary Figure 7

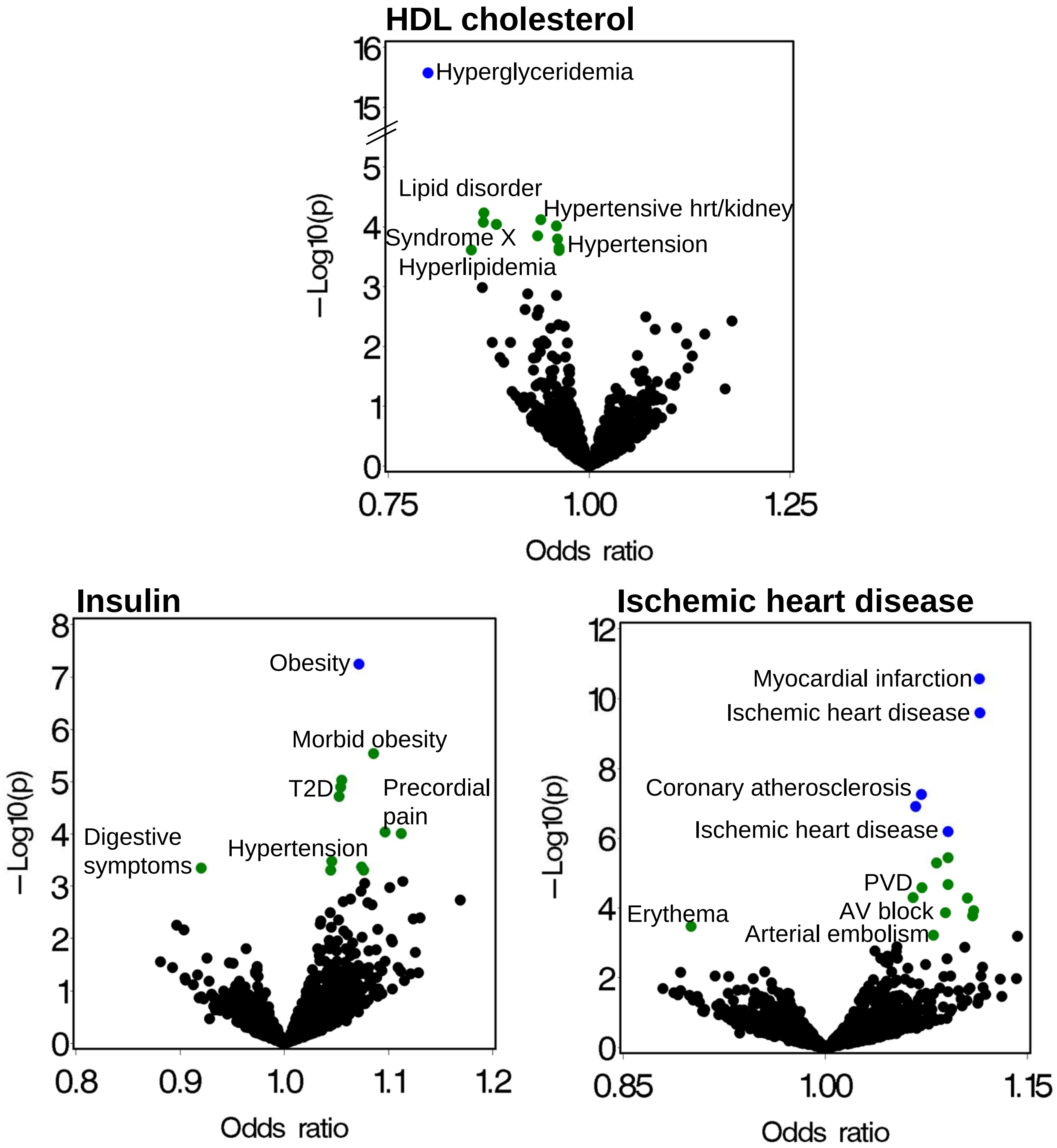

Supplementary Figure 7: Associations with metabolic biomarkers. The scatter plots summarize PheWAS analyses for genetic predictors of ARIC biomarkers. Odds ratios are from logistic regression analyses, adjusting for birth decade, gender and 3 principal components, and represent the risk associated with a 1 standard deviation change in the value of the genetic predictor. Points highlighted in blue and green are significant at Bonferroni $p<0.05$ and FDR $q<0.1$, respectively. PVD=peripheral vascular disease; IHD=ischemic (coronary) heart disease; T2D= type 2 diabetes.

Supplementary Figure 8

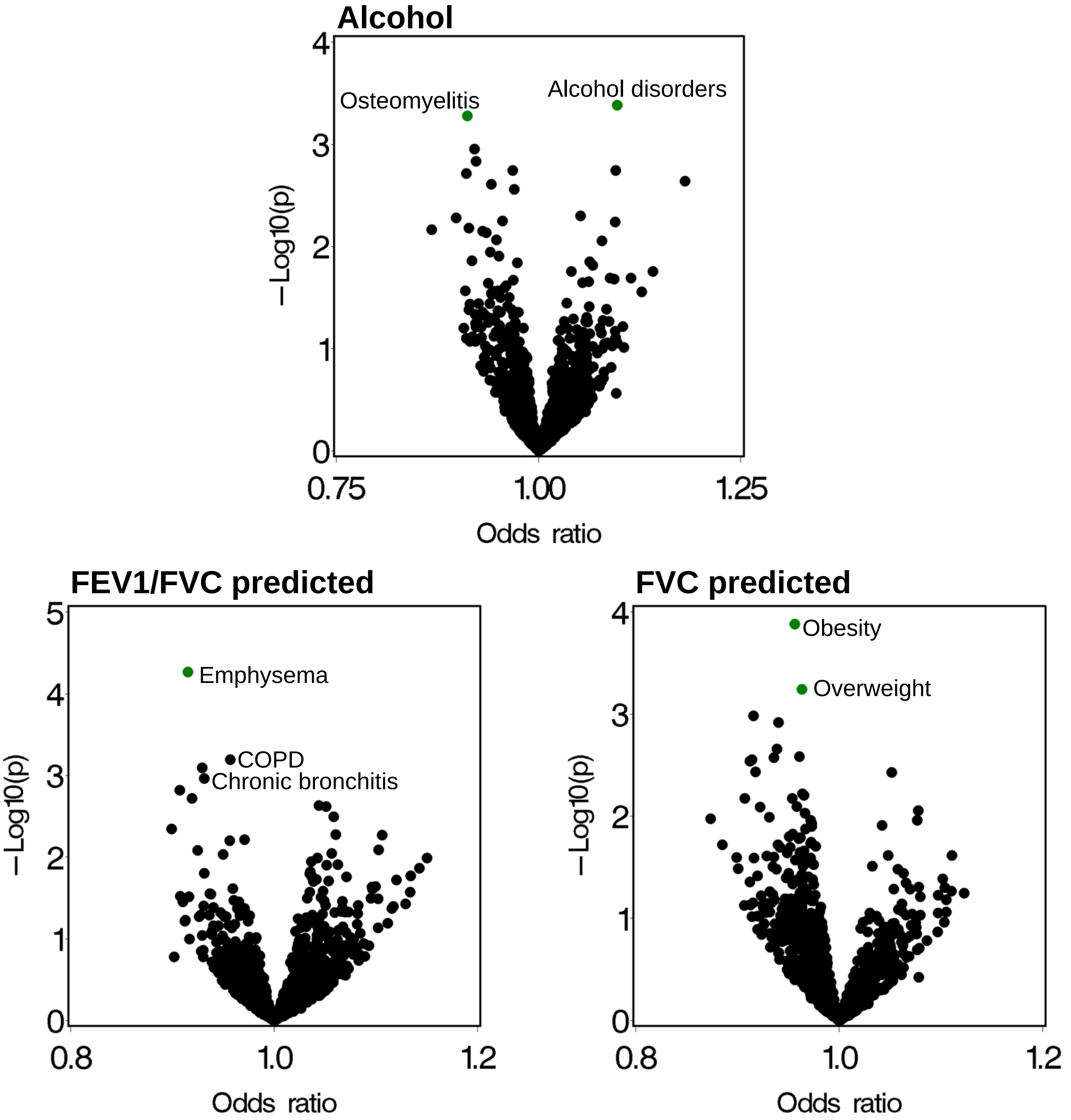

Supplementary Figure 8: Associations with alcohol and pulmonary function biomarkers. The scatter plots summarize pheWAS analyses for genetic predictors of ARIC biomarkers. Odds ratios are from logistic regression analyses, adjusting for birth decade, gender and 3 principal components, and represent the risk associated with a 1 standard deviation change in the value of the genetic predictor. Points highlighted in green are significant at FDR q<0.1. FEV1=forced expiratory volume, 1 second; FVC=forced vital capacity, COPD=chronic obstructive pulmonary disease.

Supplementary Figure 9

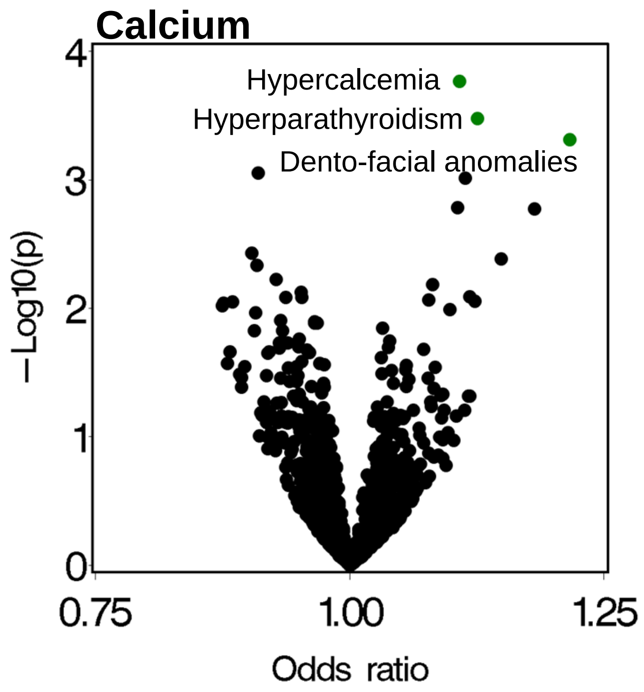

Supplementary Figure 9: Associations with serum calcium. The scatter plot summarizes PheWAS analyses for genetic predictors of ARIC biomarkers. Odds ratios are from logistic regression analyses, adjusting for birth decade, gender and 3 principal components, and represent the risk associated with a 1 standard deviation change in the value of the genetic predictor. Points highlighted in green are significant at FDR q<0.1.

Supplementary Figure 10

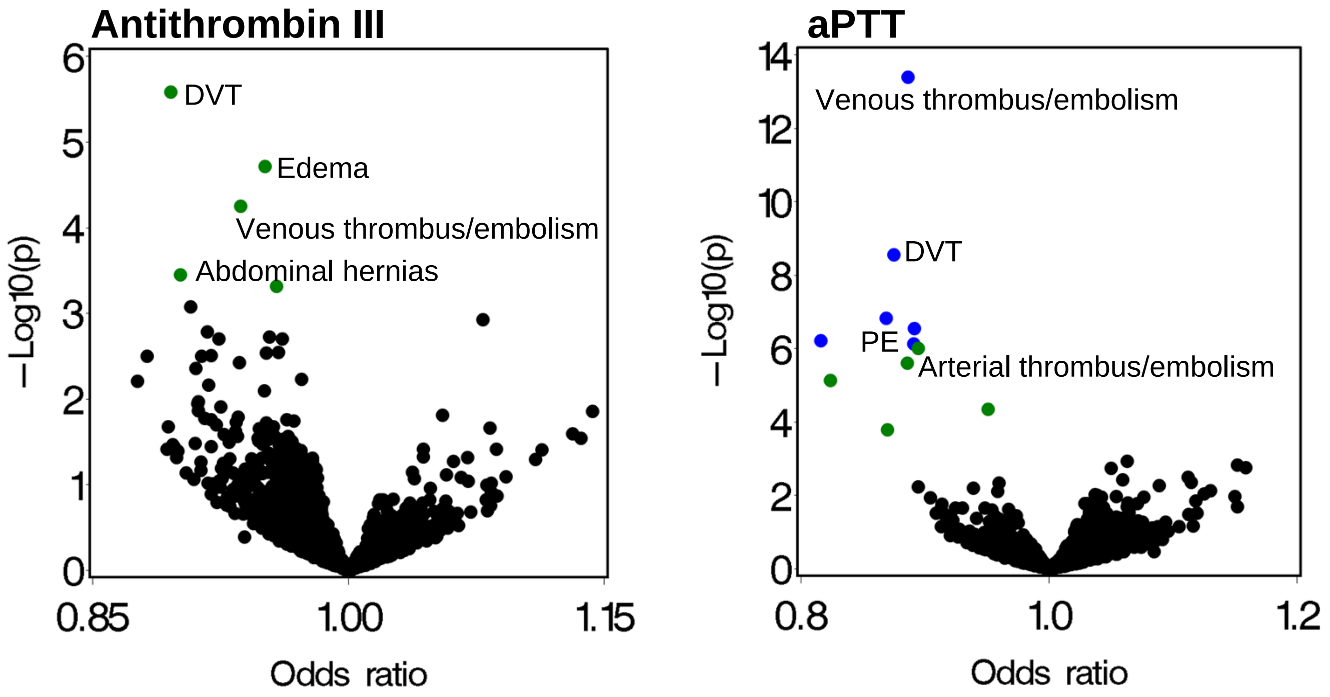

Supplementary Figure 10: Associations with coagulation biomarkers. The scatter plots summarize pheWAS analyses for genetic predictors of ARIC biomarkers. Odds ratios are from logistic regression analyses, adjusting for birth decade, gender and 3 principal components, and represent the risk associated with a 1 standard deviation change in the value of the genetic predictor. Points highlighted in blue and green are significant at Bonferroni $p<0.05$ and FDR $q<0.1$, respectively. DVT=deep vein thrombosis; PE=pulmonary embolism.

Supplementary Figure 11

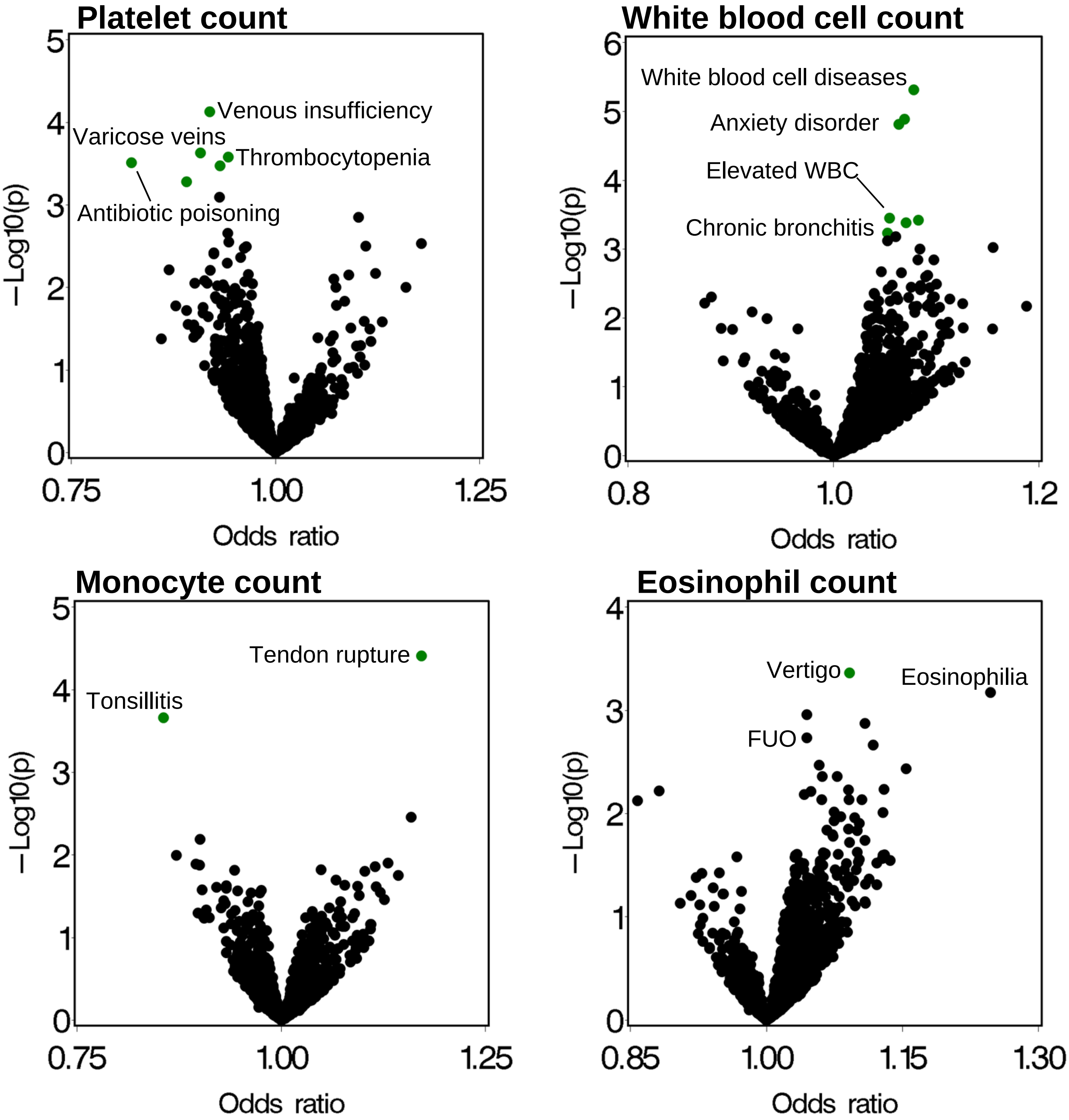

Supplementary Figure 11: Associations with hematological biomarkers. The scatter plots summarize pheWAS analyses for genetic predictors of ARIC biomarkers. Odds ratios are from logistic regression analyses, adjusting for birth decade, gender and 3 principal components, and represent the risk associated with a 1 standard deviation change in the value of the genetic predictor. Points highlighted in green are significant at FDR q<0.1. WBC=white blood cell; FUO=Fever of unknown origin.

Supplementary Figure 12

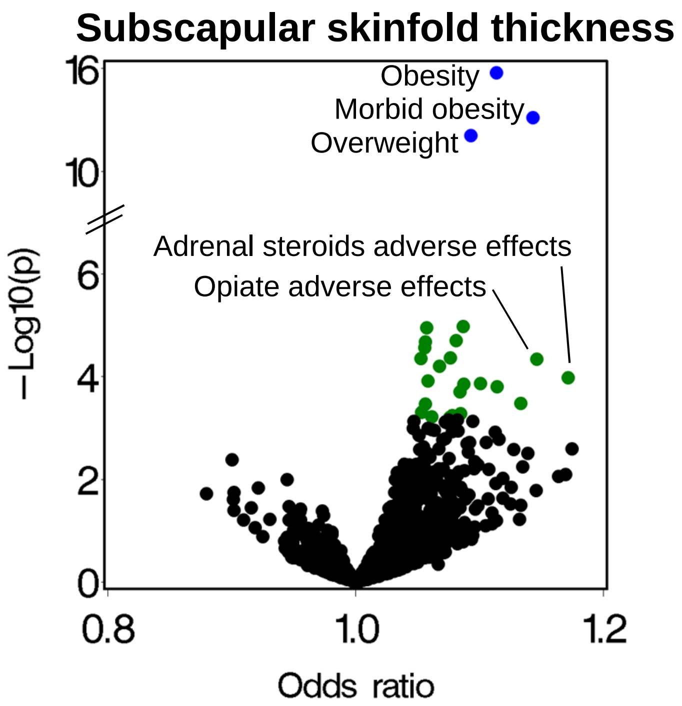

Supplementary Figure 12: Associations with subscapular skinfold thickness. The scatter plots summarize PheWAS analyses for genetic predictors of ARIC phenotypes. Odds ratios are from logistic regression analyses, adjusting for birth decade, gender and 3 principal components, and represent the risk associated with a 1 standard deviation change in the value of the genetic predictor. Points highlighted in blue and green are significant at Bonferroni p<0.05 and FDR q<0.1, respectively.

Supplementary Figure 13

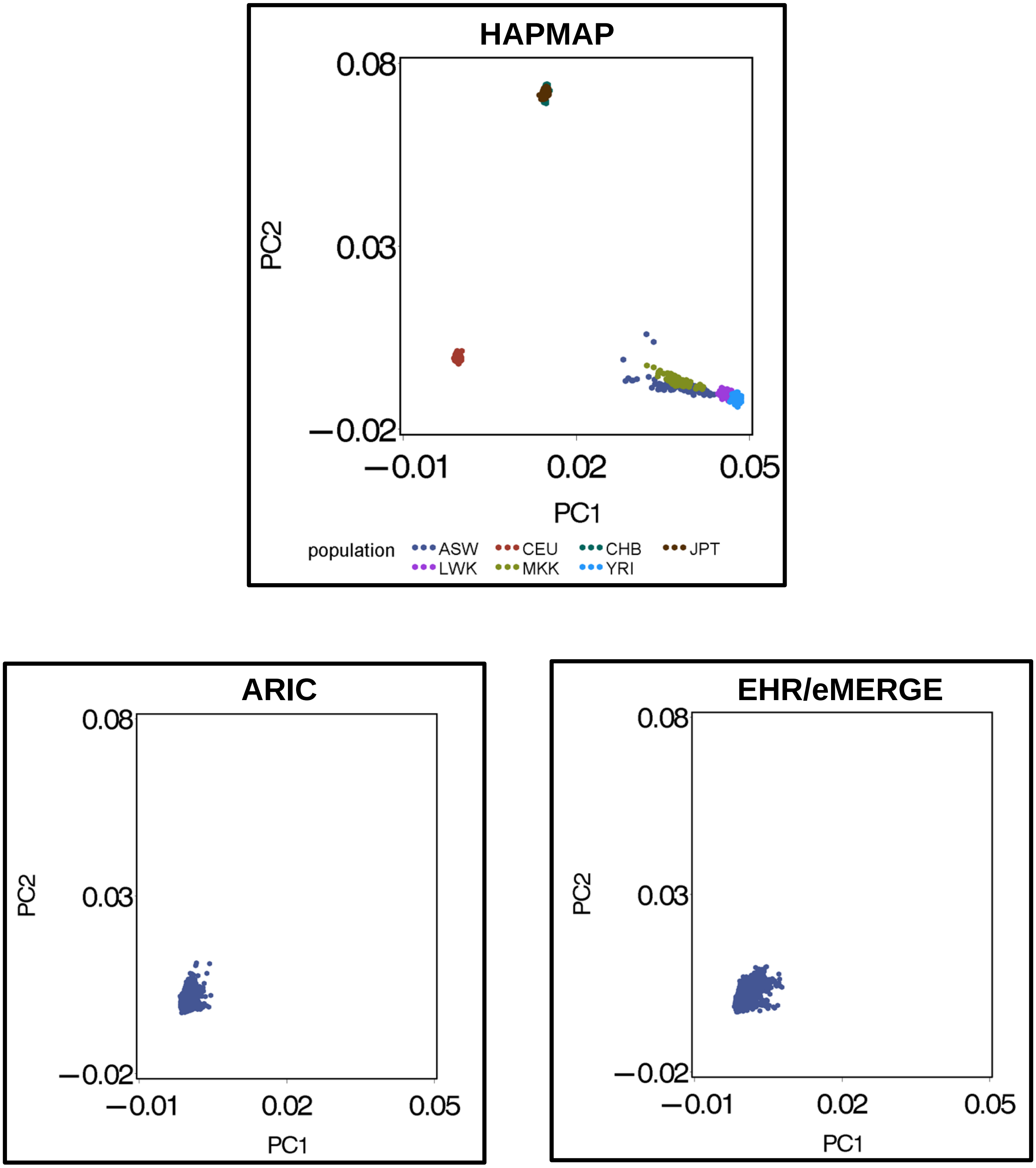

Supplementary Figure 13: Visualization of the ARIC and EHR populations by principal components. The scatter plots of PC1 and PC2 for HAPMAP references populations, the ARIC population and the EHR population.

Supplementary Table 1: Characteristics of the ARIC population.

Characteristic	Value
**Sex [n (%)]**	
Males	3,649 (47.1)
Females	4,091 (52.9)
**Age at first visit (years)**	
median (IQR)	54 (49-59)
**Biomarker phenotypes [Mean (s.d)]**	
Alcohol intake	1.2 (1.6)
Ankle-brachial index (ABI)	1.1 (0.1)
Anti-thrombin III	110.1 (20.9)
Apolipoprotein A	7.2 (0.2)
Apolipoprotein B	6.8 (0.3)
Basophil count	0.6 (1.2)
Blood urea nitrogen	2.7 (0.2)
Body mass index (BMI)	26.8 (4.6)
Carotid intima-medial thickness (CIMT), Average L bifurcation	-0.2 (0.3)
Coronary heart disease	1,325 cases
Diastolic blood pressure (DBP)	0.1 (8.9)
Eosinophil count	2.7 (0.6)
Factor VII level	4.7 (0.2)
Factor VIIIc level	4.8 (0.3)
FEV1/FVC, predicted	0.9 (0.1)
Fibrinogen level	5.7 (0.2)
Forced expiratory volume 1s (FEV1)	0.9 (0.2)
Forced vital capacity (FVC)	1.0 (0.1)
Height	168.9 (9.5)
Hematocrit level	42.2 (3.7)
Hemoglobin level	14.1 (1.3)
High density lipoprotein (HDL) cholesterol	0.2 (0.3)
High sensitivity c-reactive protein (hsCRP)	0.8 (1.1)
Low density lipoprotein (LDL) cholesterol	3.5 (0.9)
Lymphocyte count	5.2 (0.3)
Mean cellular hemoglobin (MCH)	31.1 (1.6)
Mean corpuscular volume (MCV)	90.7 (4.1)
Monocyte count	3.5 (0.5)
Neutrophil count	5.9 (0.4)
Non-HDL cholesterol	4.2 (1.1)
Partial thromboplastin time (aPTT)	3.4 (0.1)

Platelet count	5.5 (0.2)
RBC distribution width (RDW)	1.1 (0.0)
Serum calcium	9.8 (0.4)
Serum creatinine	0.1 (0.2)
Serum Glucose	1.7 (0.1)
Serum Insulin	4.1 (0.7)
Serum Magnesium	1.7 (0.1)
Serum phosphorous	3.4 (0.5)
Serum Potassium	4.5 (0.5)
Serum protein C	3.2 (0.6)
Serum triglycerides (Tg)	0.3 (0.5)
Serum uric acid	5.9 (1.5)
Smoking	343.9 (438.0)
Subscapular skinfold thickness	21.8 (9.3)
Systolic blood pressure (SBP)	0.0 (16.4)
Total cholesterol	5.5 (1.0)
Total protein	7.2 (0.4)
Type 2 diabetes (T2D)	1,335 cases
Von Willebrand factor (vWF)	111.5 (42.8)
Waist circumference	95.3 (13.5)
Waist-hip ratio (WHR)	0.9 (0.1)
White blood cell count	1.8 (0.3)

Supplementary Table 2: Positive control pairs.
For each biomarker phenotype, 1 or more pheWAS codes closely related to the phenotype were identified, prior to performing the pheWAS analysis. The pairings were classified as either "Disease defining" or "Biomarker", as indicated in the Methods. The rank is the position of the phenotype on the list of pheWAS associations that has been sorted by p-value (for instance, a rank of 1 indicates that the phenotype was the most significant association [measured by p-value] for the pheWAS, and a rank of 6 indicates that the phenotype was the 6th most significant association). The categories are used to computed tallies based on either an FDR p-value threshold or a rank threshold.

ARIC Biomarker	Biomarker type	Positive control pheWAS phenotype(s)	Phenotype rank	Bonferroni p-value	Rank category	FDR p-value
Ankle-brachial index (ABI)	Biomarker	PVD	155	>=0.05	>5	>=0.1
Anti-thrombin III	Biomarker	DVT, Arterial thrombus	1	>=0.05	<=05	<0.05
Apolipoprotein B	Biomarker	Hyperlipidemia	2	<0.05	<=05	<0.05
Factor VII level	Biomarker	DVT, Arterial thrombus	296	>=0.05	>5	>=0.1
Factor VIIIc level	Biomarker	DVT, Arterial thrombus	2	<0.05	<=05	<0.05
FEV1/FVC, predicted	Biomarker	Chronic airway obstruction	2	>=0.05	<=05	>=0.1
Fibrinogen level	Biomarker	Defibrination syndrome	5	>=0.05	<=05	>=0.1
Mean corpuscular volume (MCV)	Biomarker	Iron def anemias, Megaloblastic anemia	3	<0.05	<=05	<0.05
Partial thromboplastin time (aPTT)	Biomarker	DVT, Arterial thrombus	2	<0.05	<=05	<0.05
Serum creatinine	Biomarker	CKD	65	>=0.05	>5	>=0.1
Serum Insulin	Biomarker	T2D	4	>=0.05	<=05	<0.05
Serum protein C	Biomarker	DVT, Arterial thrombus	114	>=0.05	>5	>=0.1
Serum uric acid	Biomarker	Gout	1	>=0.05	<=05	<0.05
Subscapular skinfold thickness	Biomarker	Obesity	1	<0.05	<=05	<0.05
Von Willebrand factor (vWF)	Biomarker	DVT, Arterial thrombus	2	<0.05	<=05	<0.05
Waist circumference	Biomarker	Obesity	2	<0.05	<=05	<0.05
Waist-hip ratio (WHR)	Biomarker	Obesity	2	<0.05	<=05	<0.05
Alcohol intake	Disease defining	Alcohol disorders	1	>=0.05	<=05	<0.1
Body mass index (BMI)	Disease defining	Obesity	1	>=0.05	<=05	<0.05
Carotid intima-medial thickness (CIMT), Average L bifurcation	Disease defining	Carotid stenosis	101	>=0.05	>5	>=0.1
Coronary heart disease	Disease defining	CAD	3	<0.05	<=05	<0.05
Diastolic blood pressure (DBP)	Disease defining	Essential hypertension	2	<0.05	<=05	<0.05
Eosinophil count	Disease defining	Eosinophilia	2	>=0.05	<=05	>=0.1
Hematocrit level	Disease defining	Iron def anemias, Megaloblastic anemia	586	>=0.05	>5	>=0.1
Hemoglobin level	Disease defining	Iron def anemias, Megaloblastic anemia	3	<0.05	<=05	<0.05
High sensitivity c-reactive protein (hsCRP)	Disease defining	Elevated CRP	2	>=0.05	<=05	>=0.1
Low density lipoprotein (LDL) cholesterol	Disease defining	Hyperlipidemia	2	<0.05	<=05	<0.05
Neutrophil count	Disease defining	Neutropenia	394	>=0.05	>5	>=0.1
Non-HDL cholesterol	Disease defining	Hyperlipidemia	2	<0.05	<=05	<0.05
Platelet count	Disease defining	Thrombocytopenia, Polycythemia Vera, secondary	3	>=0.05	<=05	<0.1
Serum calcium	Disease defining	Hypercalcemia, Hypocalcemia	1	>=0.05	<=05	<0.05
Serum Glucose	Disease defining	Abnormal glucose	2	<0.05	<=05	<0.05
Serum Magnesium	Disease defining	Mg disorders	1	>=0.05	<=05	<0.05
Serum phosphorous	Disease defining	PO4 disorders	194	>=0.05	>5	>=0.1
Serum Potassium	Disease defining	Hyperpotassemia, Hypopotassemia	545	>=0.05	>5	>=0.1
Serum triglycerides (Tg)	Disease defining	Hyperglyceridemia	1	<0.05	<=05	<0.05
Smoking	Disease defining	Tobacco user	1	<0.05	<=05	<0.05
Systolic blood pressure (SBP)	Disease defining	Essential hypertension	2	<0.05	<=05	<0.05
Total cholesterol	Disease defining	Hypercholesterolemia	1	<0.05	<=05	<0.05
Total protein	Disease defining	Plasma protein metabolism d/o	940	>=0.05	>5	>=0.1
Type 2 diabetes (T2D)	Disease defining	T2D	1	<0.05	<=05	<0.05
White blood cell count	Disease defining	Diseases of WBC, Decreased WBC count, Elevated WBC count	1	>=0.05	<=05	<0.05

Supplementary Table 3: Associations between select PheWAS diagnoses with a genetic predictor of LDL, stratified by T2D status.

Group	PheWAS code	PheWAS phenotype	Cases	Controls	OR	95% CI	p-Value
All Subjects	38.3	Bacteremia	1,966	30,062	0.91	(0.87 - 0.95)	2.7E-05
Subjects with T2D	38.3	Bacteremia	1,014	8,808	0.87	(0.82 - 0.93)	3.6E-05
Subjects without T2D	38.3	Bacteremia	952	21,254	0.95	(0.89 - 1.02)	0.14
All Subjects	38	Septicemia	3,844	30,062	0.93	(0.90 - 0.96)	4.6E-05
Subjects with T2D	38	Septicemia	1,870	8,808	0.91	(0.87 - 0.96)	5.3E-04
Subjects without T2D	38	Septicemia	1,974	21,254	0.96	(0.91 - 1.00)	0.057

Supplementary Table 4: Summary of associations between Low versus Normal LDL-C and either the Bacteremia or Septicemia PheWAS phenotypes. All associations are adjusted for age, gender and self-reported race.

Type 2 diabetics (n=1,392)

Phenotype	Cases	Controls	Odds-ratio	95% CI	p-value
**Septicemia**	102	1,220	2.03	(1.23 - 3.35)	5.9E-03
**Bacteremia**	52	1,220	2.30	(1.18 - 4.45)	1.4E-02

Non-diabetics (n=20,889)

Phenotype	Cases	Controls	Odds-ratio	95% CI	p-value
**Septicemia**	369	19,987	3.76	(2.93 - 4.79)	< 2E-16
**Bacteremia**	172	19,987	4.07	(2.85 - 5.71)	1.9E-15

All subjects (n=22,281)

Phenotype	Cases	Controls	Odds-ratio	95% CI	p-value
**Septicemia**	471	21,207	3.54	(2.81 - 4.46)	< 2E-16
**Bacteremia**	224	21,207	3.60	(2.65 - 4.90)	< 2E-16

Supplementary Table 5: Characteristics of the EHR populations.

Characteristic	European ancestry primary analyses (n=37,153)	Feasibility study in self-reported blacks (n=8,552)
**Sex [n (%)]**		
Males	19,330 (52.0)	2,784 (32.6)
Females	17,823 (48.0)	5,768 (67.4)
**Birth Decade**		
median (IQR)	1945 (1935-1955)	1965 (1955-1985)
**Contributing center [n (%)]**		
Marshfield	3,707 (10.0)	n/a
VUMC	20,230 (54.5)	3,506 (41.0)
Group Health	2,358 (6.4)	114 (1.3)
Mayo	6,665 (17.9)	n/a
Northwestern	1,247 (3.4)	586 (6.9)
Geisinger	2,946 (7.9)	n/a
Harvard	n/a	249 (2.9)
Mt. Sinai	n/a	3,757 (43.9)
CCHMC	n/a	33 (0.4)
CHOP	n/a	307 (3.6)

Supplementary Table 6: Genotyping platforms and counts for the EHR subjects.

PlinkSet	Platform	Count	Percent
eMERGE I sites	Human660W-Quadv1_A	14,196	38.21
Geisinger	HumanOmniExpress-12v1.0	2,946	7.93
MarshField	Affymetrix6.0	559	1.5
Mayo	IlluminaHuman-610 and -550	3,051	8.21
VUMC	MEGA-EX_Consortium_v2_15070954_A1_b138	6,171	16.61
VUMC	HumanOmniExpressExome-8v1.2A	4,960	13.35
VUMC	HumanOmni1-QUAD	3,962	10.66
VUMC	HumanOmni5-QUAD	1,308	3.52

Supplementary Table 7: Characteristics of the LDL-C epidemiological cohort.

Characteristic	Low LDL Cohort (N=2,070)	Normal LDL Cohort (N=20,211)
Age (years) [mean, s.d.]	43.9 ± 17.5	46.4 ± 15.5
Female	1,156 (55.8%)	11,714 (58.0%)
Male	912 (44.1%)	8,476 (41.9%)
Unknown	2 (0.1%)	21 (0.1%)
Self-reported race:		
White	1,309 (63.2%)	14,448 (71.5%)
Black	386 (18.7%)	2,352 (11.6%)
Other	375 (18.1%)	3,411 (16.9%)
BMI	27.6 ± 7.4	28.8 ± 6.9
